# Leveraging *Saccharum officinarum* for an Exquisite Symmetric Supercapacitor—A Cost-Effective MnCO_3_ Synthesis Approach for Energy Storage Application

**DOI:** 10.1021/acs.jpclett.5c02141

**Published:** 2025-09-09

**Authors:** Joel Skaria Joseph, Jeyakiruba Palraj, Subramanian Sakthinathan, Helen Annal Therese, Te-Wei Chiu

**Affiliations:** ‡ Department of Materials and Mineral Resources Engineering, 34877 National Taipei University of Technology, No. 1, Section 3, Chung-Hsiao East Road, Taipei 106, Taiwan; § Institute of Materials Science and Engineering, 34877 National Taipei University of Technology, No. 1, Section 3, Chung-Hsiao East Road, Taipei 106, Taiwan; ∥ Futuristic Energy Storage Technology Lab (FESTL), Department of Chemistry, Faculty of Engineering and Technology, 93104 SRM Institute of Science and Technology, Kattankulathur, Chennai 603203, India

## Abstract

Sugarcane (*Saccharum officinarum*) was employed as a sustainable carbon source to synthesize three-dimensional (3D) spherical manganese carbonate (MnCO_3_) microspheres, offering a green route to advanced electrode material for high-energy-density symmetric supercapacitors. Although numerous synthesis strategies and material modifications have been explored, a detailed evaluation of environmentally friendly synthesis pathways remains essential. In this study, MnCO_3_ microspheres were successfully synthesized via a sugar-derived green synthesis followed by hydrothermal treatment. Owing to their distinctive morphology and tunable structure, MnCO_3_ electrodes outperformed several conventional metal oxides and hydroxides. Electrochemical studies in a three-electrode configuration under alkaline conditions demonstrated a specific capacitance of 366 F/g at 0.7 A/g and an excellent cycling stability, with 99% coulombic efficiency after 3000 cycles. Upon configuration into a symmetric supercapacitor device, the electrode operated at a high voltage of 1.2 V, delivering a specific capacitance of 179.8 F/g at 0.5 mA while retaining 50 F/g at 3 mA. The device exhibited an outstanding durability with 99.6% coulombic efficiency over 10 000 cycles and achieved a high energy density of 35.9 Wh/kg and a power density of 2590.6 W/kg. These findings highlight the potential of sugar-cane-derived 3D MnCO_3_ microspheres as cost-effective, ecofriendly electrode materials for next-generation sustainable energy storage systems.

## Graphical abstract

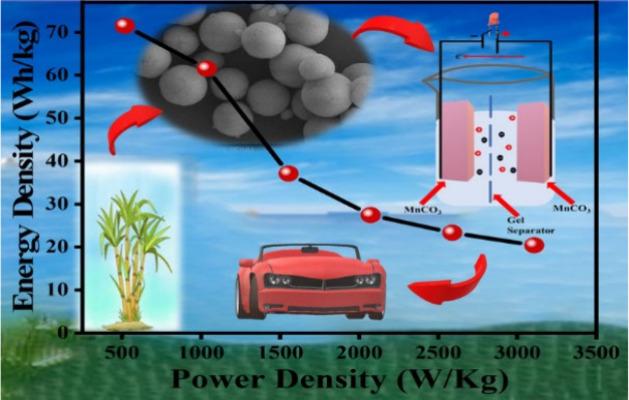

The escalating environmental crises, including global warming and climate change to food and energy shortages, have emphasized the urgent need for the development of reliable and efficient energy storage systems especially toward a greener technology to support the growing demand for sustainable and renewable energy sources.^1–4^ Among various renewable resources, sugar cane (*Saccharum officinarum*), a fast-growing grass, has emerged as a promising candidate attracting significant attention. Sugar cane, a tall tropical grass that can grow up to 20 ft, is typically harvested by cutting the tops while leaving the stalks intact, allowing it to regenerate without replanting. This makes it a highly renewable resource. Here, sugar derived from sugar cane is employed as a carbon source, offering an alternative to commonly used pyrrole for the synthesis of three-dimensional (3D) MnCO_3_ spheres with high surface area, aimed at enhancing energy storage performance.

Among the various materials explored for supercapacitor electrodes, manganese carbonate has emerged as a fascinating candidate, competing strongly with metal oxides and hydroxides due to its unique combination of properties, like high theoretical capacitance, environmental friendliness, structural versatility, and redox-active behavior.^5–7^ In general, manganese-based compounds are well-known in the field of energy storage, including batteries, supercapacitors, and fuel cells, owing to their high abundance, low cost, and eco-friendliness.^6^ Specifically, MnCO_3_ exhibits excellent pseudocapacitive behavior, where the charge storage occurs through reversible faradaic reactions, resulting in high specific capacitance values.^6–8^

A variety of MnCO_3_ microstructures with diverse morphologies have been employed in the energy storage field. Vardhan et al. synthesized uniform mesoporous MnCO_3_ by the co-precipitation method, achieving a maximum specific capacitance of 144 F/g at 0.34 A/g with 93% capacity retention over 1000 cycles.^9^ In comparison, MnCO_3_ synthesized on Ni foam by Peng et al. also delivered a lower capacitance value spanning a similar number of cycles with 9% capacity reduction.^10^ In a different study, Zhang et al. reported the synthesis of MnCO_3_ nanospheres using benign solvents and mild conditions, which offer a lower environmental impact, and demonstrated a specific capacitance of 129 F/g at 0.15 A/g.^11^ Likewise, Udayabhanu et al. reported a tea-extract-based one-pot green synthesis of MnCO_3_–rGO composites. The complex plant extracts rich in diverse polyphenols and other phytochemicals led to uneven particle morphology, chemical control issues, and variations in electrochemical behavior.^12^ Also, Kesavan et al. employed a template-assisted hydrothermal method to obtain hollow MnCO_3_ spheres for lithium-ion battery (LIB) anodes, delivering an initial capacity of 330 mAh/g at a current density of 44 mA/g in the first cycle. The capacity decreased drastically in subsequent cycles, dropping from 85 to 64 mAh/g and further down to 27.8 mAh/g after 50 cycles.^13^ Sucrose-assisted hydrothermal synthesis of MnCO_3_/Mn_3_O_4_ hybrid nanostructures has demonstrated 191 F/g specific capacitance and ∼97.8% cyclic stability after 3000 cycles in a three-electrode arrangement.^14^ Similarly, a green protocol reported by Zhang et al. emphasized sustainable generation of MnCO_3_ along with the recovery of byproducts, like (NH_4_)_2_SO_4_, underlining environmental benefits.^15^ Li et al. also achieved controlled hydrothermal growth of uniform cubic MnCO_3_ crystals using sucrose and tartaric acid, giving materials with strong Li^+^ adsorption capabilities.^16^

In the present work, we report the synthesis of MnCO_3_ using a green approach, wherein sugar is derived from sugar cane serves as a natural reducing agent. It is formed via a process that starts with the nucleation of MnCO_3_ nanospheres, which progressively come together to form bigger assemblies. Ostwald ripening is the process by which smaller, less stable particles dissolve and their substance redeposits onto bigger, more stable ones as the reaction progresses.^5^ Synthesized MnCO_3_ was directly coated onto a graphite sheet and evaluated as an electrode material for supercapacitor applications. Electrochemical measurements conducted in a 2 M KOH electrolyte revealed a high specific capacitance of 366 F/g at a current density of 0.7 A/g. Furthermore, the electrode exhibited excellent cycling stability, retaining 98.3% of its initial capacitance with a coulombic efficiency of 99% after 3000 charge and discharge cycles at 4.2 A/g. When assembled into a symmetric device, the supercapacitor delivered a remarkable energy density of 35.9 Wh/kg and a power density of 2590 W/kg, maintaining a coulombic efficiency of 99.6% over 10 000 cycles. These findings underscore the potential of bio-assisted MnCO_3_ synthesis and its effective integration with graphite substrates, paving the way for cost-effective and high-performance energy storage devices.

Manganese carbonate (MnCO_3_) was synthesized via a hydrothermal method using potassium permanganate (0.395 g) as the manganese source and 0.5 g of sugar extracted from *S. officinarum* (sugar cane) as the carbon source and reducing agent. Both were dissolved in 10 mL of deionized water and stirred for 1 h. The resulting solution was transferred to a Teflon-lined autoclave and heated at 200 °C for 24 h. After cooling to room temperature, the precipitate was collected, thoroughly washed with deionized water and ethanol, and dried at 80 °C overnight, and finally, the product was ground into a fine powder (Figure 1).

**Figure 1:**
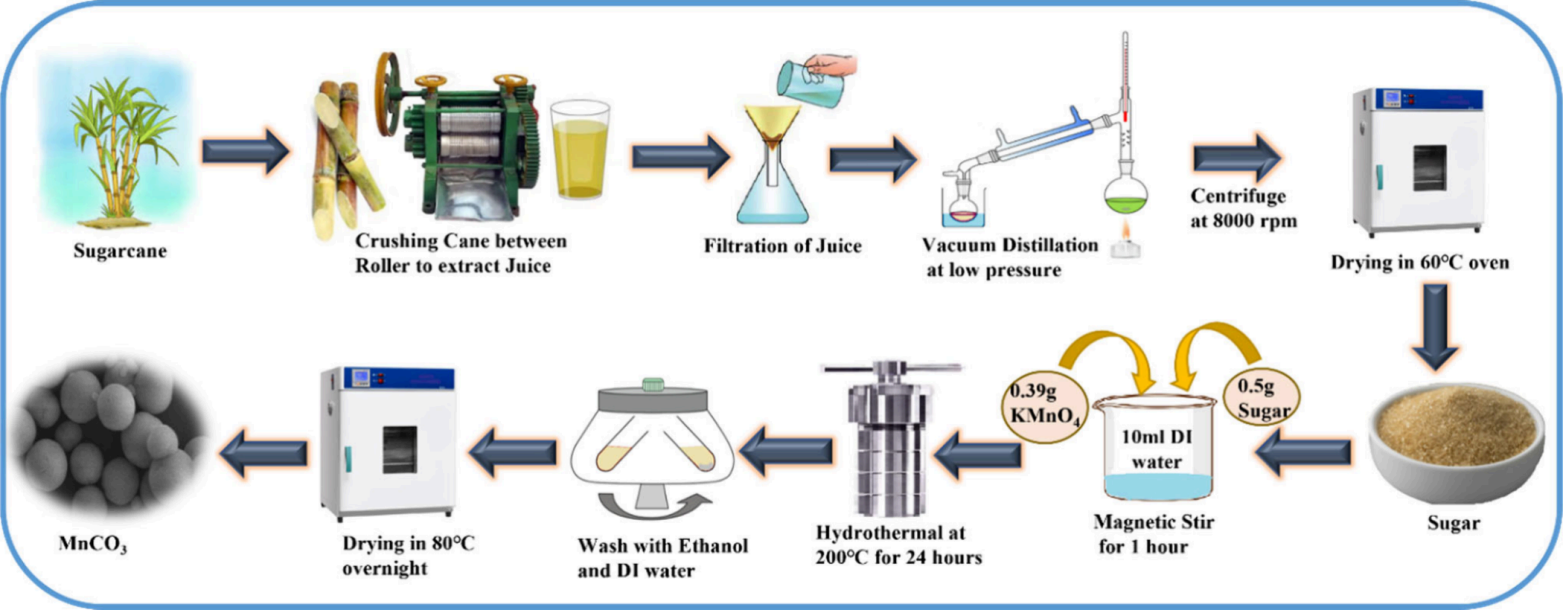
Schematic diagram of the MnCO_3_ microsphere synthesis process.

In the synthesis of the MnCO_3_ microsphere, a redox process involving *S. officinarum*-derived sugar and potassium permanganate (KMnO_4_) occurs. The sugar functions as a reducing agent, while KMnO_4_ is reduced. It reduces manganese in KMnO_4_, which is in a +7 oxidation state, to +2 (Mn^2+^) indicated by the changing of color from purple to a diminishing color, pointing toward the reduction of MnO_4_^–^. It then further oxidizes to produce carbon dioxide and water. Here, carbonate ions were introduced by allowing CO_2_ from the oxidation of sugar to dissolve congruously in water precipitating with Mn^2+^ as manganese carbonate (MnCO_3_).

MnCO_3_, a pale pink precipitate, can be extracted by the reaction between the Mn^2+^ ions brought about in the solution and the carbonate ions (CO_3_^2–^) formed from the oxidation of sugar. Carbon dioxide (CO_2_) generated in reaction [Disp-formula eqR1] dissolves in water, and through equilibrium reactions, forms carbonic acid (H_2_CO_3_), which then dissociates into bicarbonate (HCO_3_^–^) and carbonate (CO_3_^2–^), as given in the intermediate reaction [Disp-formula eqR2].

The balanced redox reaction for this synthesis is shown in reactions [Disp-formula eqR1] and [Disp-formula eqR2].

R1 $$C_{6}H_{12}O_{6}+H_{2}O\to 6{\mathrm{CO}}_{2}+24H^{+}+24e^{-}$$

R2 $${\mathrm{CO}}_{2}+H_{2}O\to H_{2}{\mathrm{CO}}_{3}\to {{\mathrm{HCO}}_{3}}^{-}+H^{+}$$

R3 $$4{\mathrm{KMnO}}_{4}+3C_{6}H_{12}O_{6}+6H_{2}O\to 4{\mathrm{MnCO}}_{3}+6{\mathrm{CO}}_{2}+K_{2}{\mathrm{CO}}_{3}+12H_{2}O$$

The X-ray diffraction (XRD) experiment was performed to assess the crystallinity and phase purity of the as-prepared 3D MnCO_3_ microspheres, as shown in Figure 2a. The diffraction peaks at 31.6°, 37.5°, 41.8°, 45.3°, 52.1°, 60.24°, 64.1, 64.1°, and 67.9° emanating from (104), (110), (113), (202), (116), (112), (214), and (300) crystallographic planes, respectively, confirm the formation of the MnCO_3_ rhombohedral phase with the space group of *R*3̅*c*.^13^ Figure 2b–d presents the high-resolution XPS spectra of Mn 2p, C 1s, and O 1s, respectively. The Mn 2p spectrum displays two distinct peaks located at 642.5 eV (Mn 2p_3/2_) and 654.0 eV (Mn 2p_1/2_), with a spin–orbit separation of approximately 11.5 eV, which is characteristic of the Mn^2+^ oxidation state in MnCO_3_.^17–19^ A minor peak observed around 647.1 eV may be attributed to Mn–O interactions, suggesting partial surface oxidation. The C 1s spectrum exhibits multiple components: the main peak at 284.8 eV corresponds to C–C or sp^2^-hybridized carbon, while peaks at 286.0 and 287.7 eV are associated with C–O species in carbonate and organic groups, respectively. A distinct peak at 290.2 eV further confirms the presence of carbonate (CO_3_^2–^) groups in the structure. The O 1s spectrum shows a dominant peak at 532.5 eV, which is also attributed to oxygen in the carbonate groups. Additionally, a peak at 534.5 eV corresponds to hydroxyl (−OH) species due to surface-bound hydroxyl groups. In this study, the morphology and microstructure of MnCO_3_ were examined using HRSEM. Figure 2e and f depicts the bulk microsphere structure as a collection of 3D spherical balls. The formation of 3D MnCO_3_ microspheres follows a mechanism that begins with the nucleation of MnCO_3_ nanospheres, which gradually aggregate into larger assemblies. As the reaction proceeds, Ostwald ripening occurs; smaller, less stable particles dissolve, and their material redeposits onto larger, more stable ones. This leads to the development of uniform 3D spherical structures. The resulting morphology features a porous framework that enhances electrolyte access and promotes rapid ion transport. These structural attributes contribute to the availability of numerous active sites for charge storage. Further detailed structural analyses were obtained through TEM analysis. As illustrated in Figure 2g–i, the TEM images reveal that these microspheres are not a solid single 3D structure but rather composed of nanostructured subunits. Individual nanodomains with sizes ranging from 200 to 500 nm are clearly visible, indicating that each microsphere is a secondary particle formed by the aggregation of smaller nanoscale building blocks.^20^ The HRTEM image with the inverse fast Fourier transform (IFFT) pattern in Figure 2i clearly shows the lattice-resolved fringes of (110) and (104) planes with a lattice spacing of 0.24 and 0.28 nm of MnCO_3_, respectively. Additional information about the morphology is given in Figure S-1. Further characterization of Raman and FTIR analyses of MnCO_3_ material supports the formation of the microsphere with the signature carbonate peak at 1078 cm^–1^, while 498 and 318 cm^–1^ wavenumber features are assigned to Mn–O lattice vibrations, indicating a well-formed rhodochrosite-like MnCO_3_ phase; also, the FTIR profile observed at 1366, 864, and 729 cm^–1^ reaffirms the presence of the carbonate group being indicative of characteristic vibrational modes of MnCO_3_, whereas 1641 cm^–1^ denotes the overtone band emerging from CO_3_^2–^ interacting with Mn^2+^. Additional material confirmation studies of Raman and FTIR analyses of MnCO_3_ are given in Figure S-3 of the Supporting Information.

**Figure 2:**
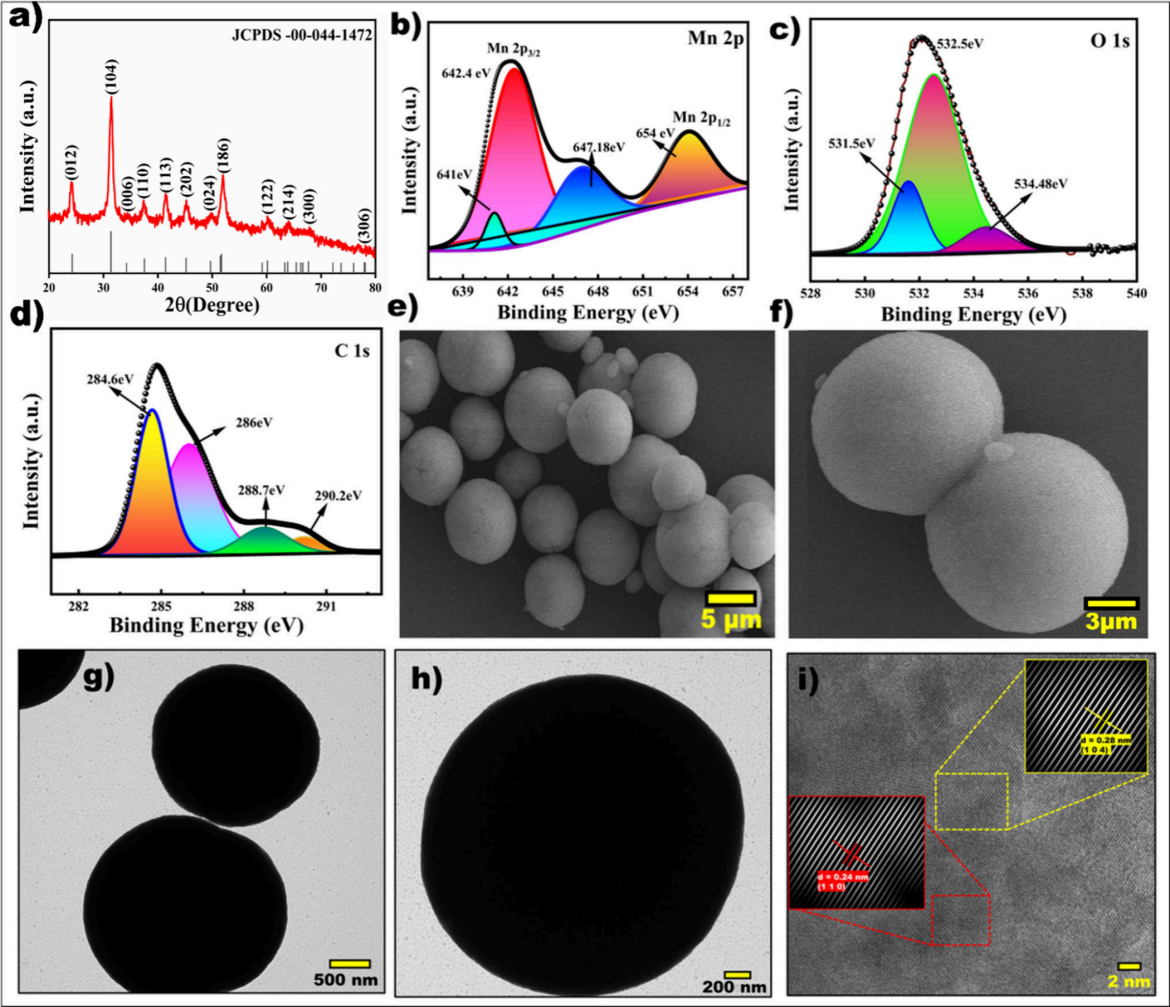
(a) XRD pattern of MnCO_3_, (b–d) XPS core spectrum of Mn 2p, O 1s, and C 1s, (e and f) FESEM images of 3D MnCO_3_ spheres, (g and h) HRTEM images at different magnifications, and (i) lattice fringes from TEM images.

The electrochemical performance of the MnCO_3_ electrode was evaluated in a 2 M KOH aqueous electrolyte, as shown in Figure 3. The working potential window was optimized between −1.0 and 0.7 V using cyclic voltammetry (CV). The CV curves at various scan rates suggested a pseudocapacitive behavior, which is displayed in Figure 3a. The bare graphite sheet analysis and comparison are discussed in Figure S-4. The voltammograms for KOH electrolytes exhibit a quasi-rectangular shape with distinct redox peaks, displaying the pseudocapacitive behavior. A similar trend can be observed in GCD curves at different current densities. All the GCD curves were recorded at various current densities of 0.7–5.0 A/g in Figure 2b.

**Figure 3:**
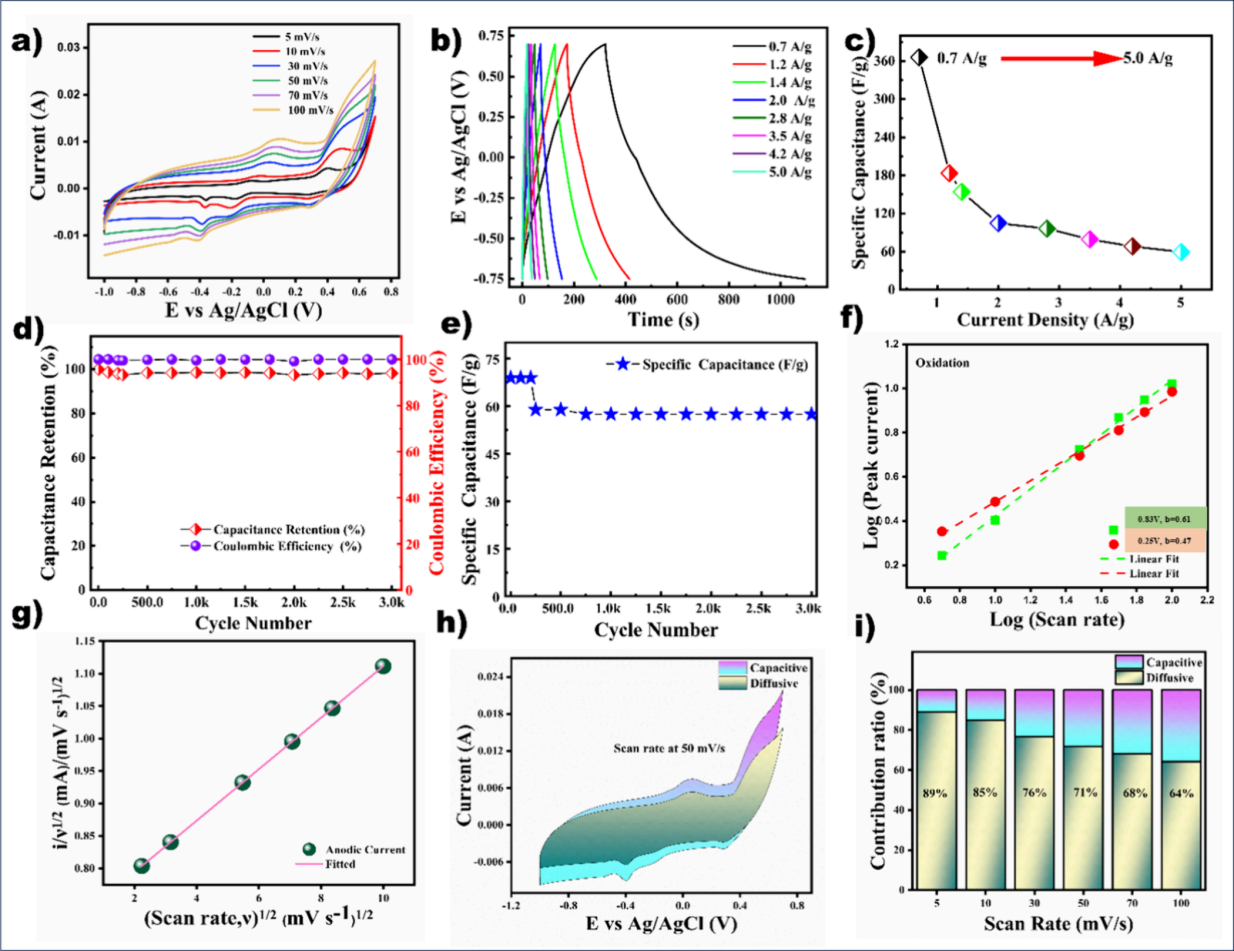
Electrochemical performance in three-electrode configurations: (a) CV curves, (b) GCD plots, (c) *C*_s_ from GCD, (d) cycling stability at 4.2 A/g, (e) cycle number vs capacitance, and (f–i) charge storage mechanism of the MnCO_3_ electrode: (f) *b* value calculation, (g) plot of *i*(*v*)/*V* ^1/2^ (A s^–1^ V^1/2^) vs (scan rate)^1/2^ (V^1/2^/s), (h) capacitive contribution in CV at 50 mV/s, and (i) capacitive/diffusion contribution in percent.

The gravimetric capacitance has been calculated using the GCD data based on eq 1 ^21,22^

1 $$C_{s}\left(F/g\right)=\frac{i\Delta t}{m\Delta V}$$

where *i* is the applied current for charge/discharge, Δ*t* is the discharge time, *m* is the electrode’s active mass, and Δ*V* is the potential window during discharge. The 3D MnCO_3_ electrodes exhibited excellent capacitance values of 366, 184, 154, 105, 97, 80, 69, and 60 F/g at current densities of 0.7, 1.2, 1.4, 2.0, 2.8, 3.5, 4.2, and 5.0 A/g, respectively, within a potential range from −0.75 to 0.7 V (Figure 3c). Long-term cycling stability was also assessed (Figure 3d). The MnCO_3_ electrode retained a specific capacitance of 69 F/g at 4.2 A/g over 3000 charge/discharge cycles, with 98% capacitance retention and 100% coulombic efficiency, demonstrating excellent electrochemical stability. Even after 3000 continuous cycles, the capacitance retained 59 F/g at a high current density of 4.2 A/g (Figure 3e), which further showcases the excellent performance of the MnCO_3_ electrode. The remarkable performance is attributed to the unique 3D spherical structure of MnCO_3_, which enhances the reversible insertion/extraction of K^+^ ions, thereby improving the charge storage capability. To learn more about the ion transport and resistance of the MnCO_3_ electrode in the 2 M KOH electrolyte, electrochemical impedance spectroscopy was analyzed. The *R*_ct_ value has drastically reduced to 9.44 Ω after cycling compared to 67.39 Ω before cycling, indicating a strong ionic kinetics and electrical conductivity, and the details are discussed in Figure S-5 of the Supporting Information.

The charge storage mechanism of the MnCO_3_ electrode was investigated using CV at different scan rates in Figure 3f–i. At specific anodic potentials of 0.83 and 0.25 V, the calculated *b* values were 0.61 and 0.47, respectively, indicating that the overall charge storage involves diffusion-controlled processes, with a significant contribution from capacitive surface properties. At a lower scan rate of 5 mV/s, the 3D MnCO_3_ electrode exhibited diffusion-dominated behavior, accounting for approximately 89% of the total current. The plot of *i*(*v*)/*V* ^1/2^ (A s^–1^ V^1/2^) vs (scan rate)^1/2^ (V^1/2^/s) was displayed in Figure 3g, which helped to find the *K*_1_ and *K*_2_ values. From these values, we observed and calculated the capacitive and diffusion behavior using CV curves.^23,24^ Figure 2h represents the 71% diffusive nature at a 50 mV/s scan rate. As shown in Figure 3i, the relative contributions of capacitive and diffusion-controlled processes were extracted from the CV curves across various scan rates. With an increasing scan rate, the capacitive contribution also progressed, rising from 11% at 5 mV/s to 36% at 100 mV/s, but diffusive remains dominant, which demonstrates the slight enhancement of capacitive behavior at higher sweep rates.

To evaluate the practical feasibility of the synthesized material, symmetric supercapacitor devices were assembled in CR2032-type coin cells using 3D MnCO_3_ electrodes coated on graphitic sheets. A PVA–KOH gel served as the solid-state electrolyte, and the electrode mass was optimized for various electrochemical performances. CV analysis revealed that the optimal operating voltage window for the device was 0–1.4 V (Figure 4a), which is a notably wide window for an aqueous-based symmetric configuration. CV curves recorded at scan rates ranging from 10 to 100 mV/s (Figure 4b) displayed well-defined redox peaks, indicating the pseudocapacitive nature of the MnCO_3_ electrode. Galvanostatic charge–discharge (GCD) measurements were conducted within a potential range of 0–1.2 V at various currents from 0.5 to 3.0 mA (Figure 4c). The resulting GCD curves exhibited nonlinear characteristics, further confirming the pseudocapacitive charge storage behavior. The specific capacitance (*C*_s_) of the device, calculated using eq 1 (Figure 4d), reached a maximum of 179.8 F/g at 0.5 mA. As the current increased, the specific capacitance values gradually decreased to 161.7, 115, 93.6, 84, and 59.8 F/g at 1, 1.5, 2, 2.5, and 3 mA, respectively. Device long-term stability was assessed through continuous cycling for 10 000 charge–discharge cycles at a current of 3 mA (Figure 4e). Impressively, the device retained 99.6% of its initial capacitance, demonstrating remarkable cycling stability and coulombic efficiency. Insets in the figures show the GCD profiles of the first and last 5 cycles, further confirming the stable operation over prolonged use. The energy and power densities were evaluated and are presented in the Ragone plot (Figure 4f).^24^ At a low current of 0.5 mA, the device delivered a high energy density of 35.96 Wh/kg along with a power density of 431.9 W/kg. The maximum recorded values for energy and power densities reached 35.96 Wh /kg and 2590.6 W/kg, respectively, surpassing several previously reported MnCO_3_-based devices. For instance, Tang et al. reported a peanut-shaped MnCO_3_ structure achieving only 14.7 Wh/kg and 90 W/kg, while Amutha et al.’s RGO–CNF–MnCO_3_ composite yielded even lower values.^25,26^ A comparative summary of the electrochemical performance with other MnCO_3_-based supercapacitors is provided in Table S-1 of the Supporting Information. These results highlight the outstanding stability, high coulombic efficiency, and enhanced energy storage capabilities of the sugar-derived MnCO_3_ electrode, emphasizing its potential for next-generation sustainable supercapacitor applications. To study further the exceptional behavior of spherical MnCO_3_ electrode, post-cycling XRD and FE-SEM were taken and the results confirm the stability of our material, also a detailed explanation is provided in Figures S-6 and S-7.

**Figure 4:**
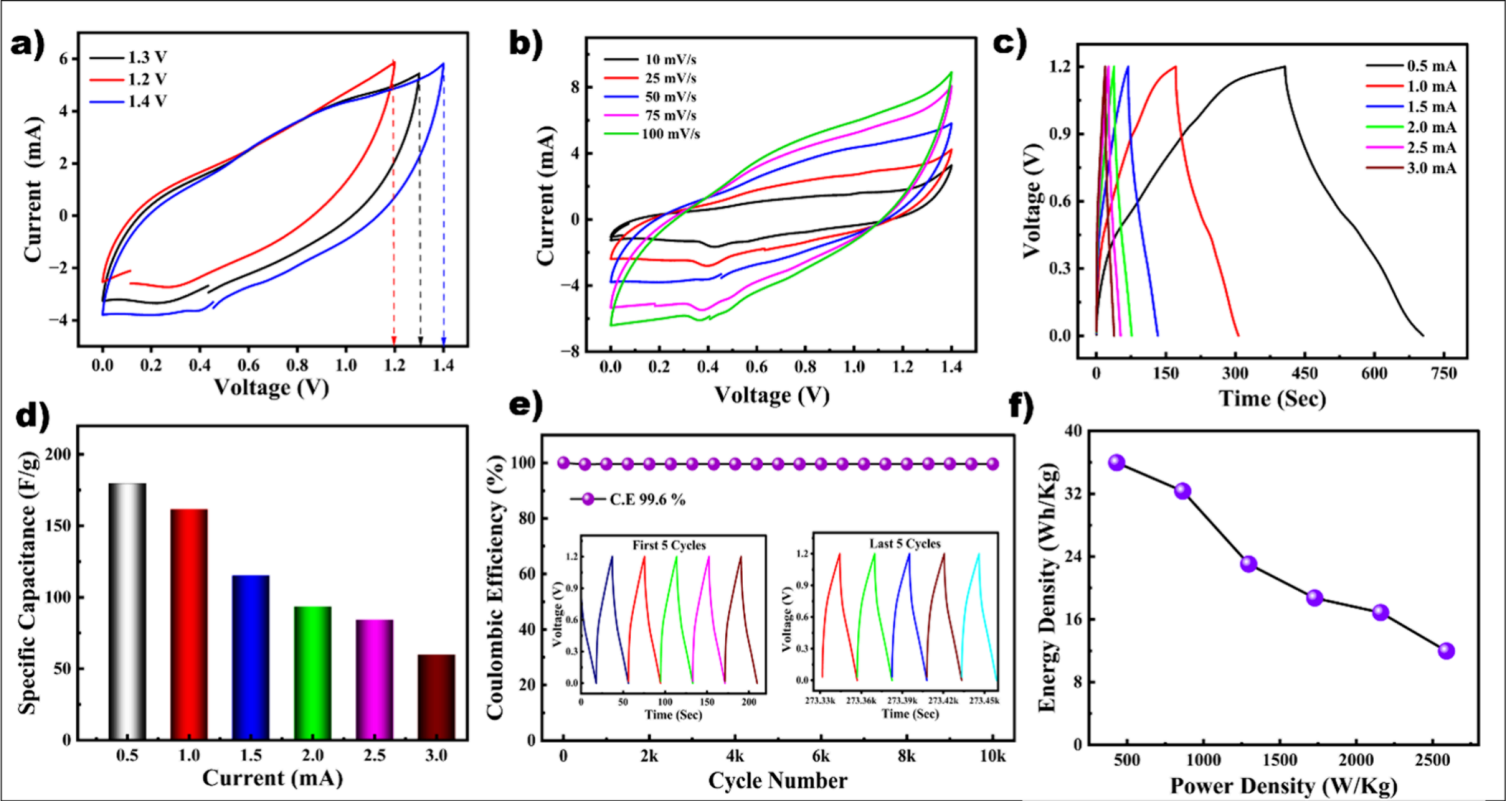
Symmetric supercapacitor device performance of 3D MnCO_3_: (a) *V* optimization, (b) CV curves, (c) GCD, (d) *C*_s_ from GCD curves, (e) long-term cycling stability with CE (inset images are the first and last 5 cycles), and (f) Ragone plot.

In summary, a facile and sustainable multi-step synthesis route was developed for the synthesis of MnCO_3_ microspheres using a sugar-derived carbon source. Structural and morphological characterizations confirmed the successful formation of well-defined 3D spherical MnCO_3_ particles. Electrochemical studies in a three-electrode system demonstrated excellent capacitive performance with a high specific capacitance of 366 F/g at 0.7 A/g and good rate capability, retaining 69 F/g at 4.2 A/g. The electrode exhibited remarkable cycling stability with 98.3% capacitance retention after 3000 cycles and a coulombic efficiency of 99%. Furthermore, the symmetric supercapacitor device based on MnCO_3_ delivered a high energy density of 35.96 Wh/kg and a maximum power density of 2590 W/kg, maintaining 99.6% coulombic efficiency over prolonged 10 000 cycles. These results demonstrate that the MnCO_3_ microspheres synthesized via this green approach offer significant promise as a cost-effective and efficient electrode material for next-generation energy storage systems.

## Supplementary Material



## Data Availability

Data will be provided upon request.

## Notes

Additional details about materials used, characterization techniques utilized, synthesis of sugar from *S. officinarum*, electrode preparation, FESEM and HRTEM analyses, Raman and FTIR spectra, three-electrode study of the bare graphite sheet, EIS study, charge storage mechanism, and comparison table of MnCO_3_-based electrodes for supercapacitor application (PDF)
