# Supplementary material for: Leveraging Saccharum officinarum for an Exquisite Symmetric Supercapacitor—A Cost-Effective MnCO3 Synthesis Approach for Energy Storage Application
Source: J Phys Chem Lett. 2025 Sep 9;16(37):9722–8. doi: 10.1021/acs.jpclett.5c02141 (PMC12451739; doi:10.1021/acs.jpclett.5c02141)
Supplement: Supplementary file 1 [file jz5c02141_si_002.pdf]

Supporting Information

Leveraging Saccharum Officinarum for exquisite symmetric supercapacitor-A cost-effective $MnCO_3$ synthesis approach for energy storage application

Joel Skaria Joseph [a,b]† , Jeyakiruba Palraj [c]†, Subramanian Sakthinathan [a,b], Helen Annal Therese [c]* , Te-Wei Chiu[a,b]*

[a] Department of Materials and Mineral Resources Engineering, National Taipei University of Technology, No. 1, Section 3, Chung-Hsiao East Road, Taipei 106, Taiwan

[b] Institute of Materials Science and Engineering, National Taipei University of Technology, No. 1, Section 3, Chung-Hsiao East Road, Taipei 106, Taiwan

[c] Futuristic Energy Storage Technology lab (FESTL), Department of Chemistry, Faculty of Engineering and Technology, SRM Institute of Science and Technology, Kattankulathur-603203, India

*Correspondence: tewei@ntut.edu.tw (Te-Wei Chiu)

helena@srmist.edu.in (Helen Annal Therese)

Materials

Potassium Permanganate ($KMnO_4$) 99% pure (SHOWA), and KOH 95% pure (EMPEROR CHEM CO), Carbon Black 99% is brought from Alfa Aesar, NMP (N-Methyl 2 Pyrrolidone) with a purity of 99% purchased from Merck and PVDF (Polyvinylidene difluoride) has purity of 99% purchased from Alfa Aesar. sugarcane is from a fruit vendor in Kerala, India.

Characterization

The configuration of the $MnCO_3$ was examined by X-ray diffraction analysis (XRD) (Bruker XRD, D2 Phaser). Field Emission Scanning Electron Microscopy (FE-SEM, FEI Quanta FEG 200, H-7600, Hitachi, Japan) operated at 200 kHz was used to analyze the structure. High-Resolution Transition Electron Microscopy (HR-TEM, JEOL Japan, JEM-2100 plus) is employed to evaluate structural investigations and selected area electron diffraction (SAED) patterns. The Omicron ESCA system, manufactured by Oxford Instruments Germany, was instrumental in X-ray photoelectron spectroscopy (XPS) to characterize the surface and chemical states. Electrochemical studies of three electrode system were done on a CHI electrochemical workstation (CHI 6273E). CR2032 coin cell was utilized for the symmetric device and Biologic/VSP300 workstation was used to perform its electrochemical analyses.

Synthesis of Sugar from Saccharum officinarum (Sugarcane Grass)

Sugarcane after harvesting is washed well with water and cut into shreds. It is then crushed using roller and water is sprayed to dissolve sucrose from the cells to obtain dilute. Lime (calcium hydroxide) is added to precipitate the impurities so they settle out and it neutralizes the acidic juice to prevent sucrose from further breaking into sucrose and fructose. The obtained juice is then filtered using a Whatman filter paper to separate out the fiber and extract only the juicy content.

The juice is then concentrated by vacuum distillation where it is heated at successive reduced pressure yet high temperature. The method is employed to get rid of water content at low pressure and high temperature else browning will take place. It is thereafter crystallized at low pressure to prevent charting and caramelization. Finally, we are separating sugar from molasses by centrifugation process at high rpm.

Electrode Preparation

Super P and PVDF (polyvinylidene fluoride) were combined with the active material, $MnCO_3$ sphere, in a mass ratio of 80:10:10. The combination above was combined with a few drops of N-Methyl 2-Pyrrolidone (NMP), which was then thoroughly mashed using an agate mortar to yield a slurry. We engaged PVDF as binder between active material and Super P whereas NMP as solvent to make slurry. This paste was then applied to a 1 cm by 1 cm graphitic sheet and dried for 12 hours at 80 °C in an oven. A mass of 1.5 mg was deposited to each electrode. Three electrodes were used for the electrochemical tests. Ag/AgCl (3M KCl) serves as the reference electrode, a platinum rod serves as the counter electrode, and a graphitic sheet covered with $MnCO_3$ nanosphere functions as the working electrode. A CHI6273 electrochemical workstation was utilized to carry out the electrochemical experiments, comprising electrochemical impedance spectroscopy (EIS), galvanostatic charge/discharge (GCD), and cyclic voltammetry (CV). The measurement for EIS was performed at an open circuit voltage with the frequency ranging from 0.1 Hz to 100 KHz and an AC amplitude of 5 mV was implemented.

FESEM & HRTEM Analysis

The shape and microstructure of the $MnCO_3$ were examined using FESEM. Figure S-1(a,b) depicts the bulk microsphere structure as collection of spherical balls. In order to comprehend the distinct shape of the $MnCO_3$ surface, lower magnification study using TEM was

done. $MnCO_3$'s sphere-like structure may be seen in HRTEM pictures Figure S-1(c,d). The structure of the nanograins gathered together to form microsphere. Looking at the elemental mapping in (Figure S-1e) we can state that the material has been properly formed with the presence of all three elements.

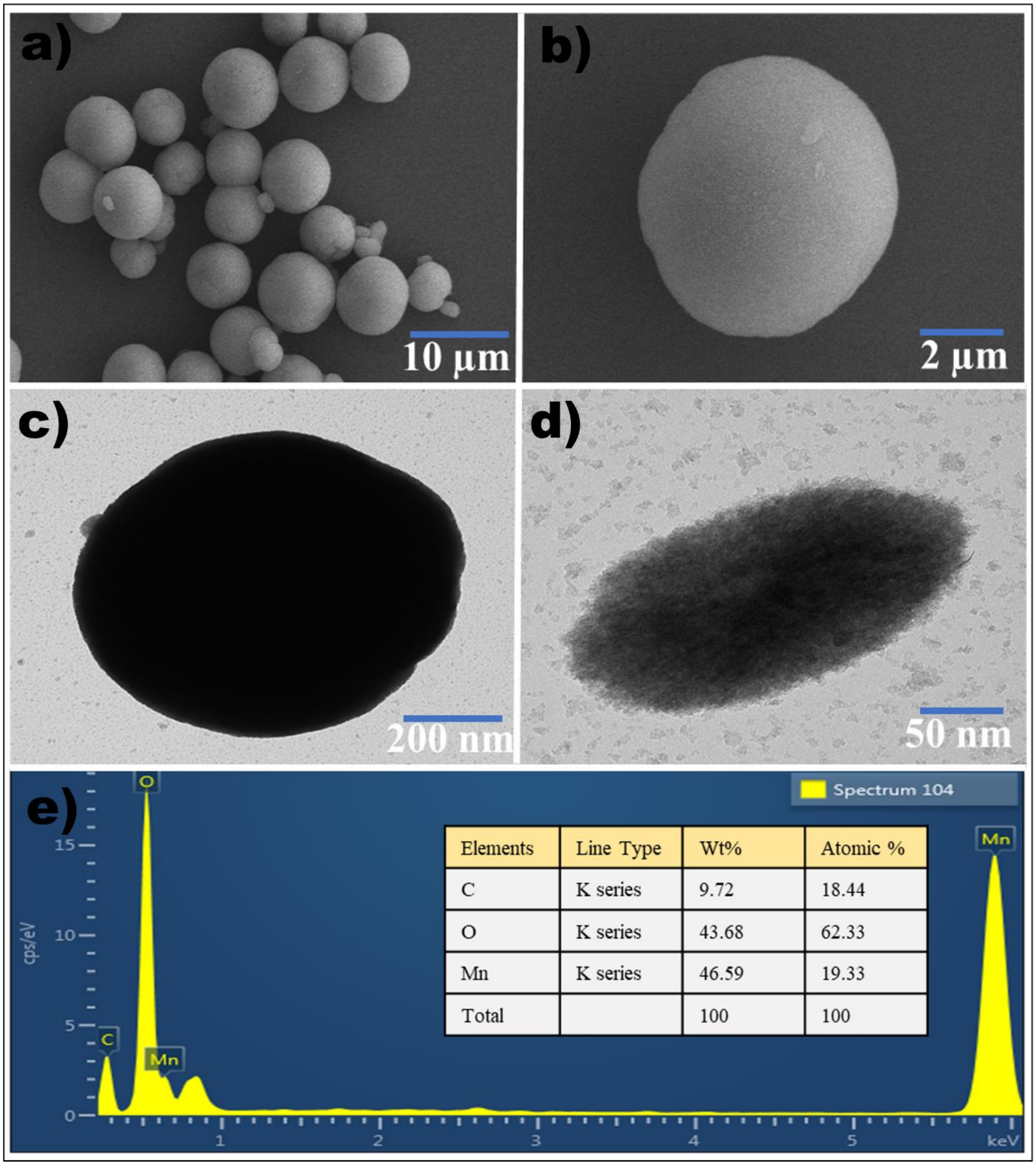

Elements	Line Type	Wt%	Atomic %
C	K series	9.72	18.44
O	K series	43.68	62.33
Mn	K series	46.59	19.33
Total		100	100

Figure S-1: FESEM images at (a) 10 µm (b) 2 µm and HRTEM images at (c) 200nm (d) 50nm magnifications, (e) Elemental Mapping of as synthesized $MnCO_3$ microsphere.

Raman Spectral study of $MnCO_3$

The material produced was characterized using Raman spectroscopy for its vibrational behavior and phase purity. The spectrum gives a series of distinct peaks at 87.96, 318.48, 498.82, 630.76, 664.08, 898.31, 1078, and 1551.9 cm^{-1}, all of which are indicative of characteristic vibrational modes of $MnCO_3$ and possible secondary phases or surface functional groups. The peaks provide essential information on the chemical structure and purity of the material. The maximum at 87.96 cm^{-1} corresponds to low-frequency lattice vibrations of rhombohedral structure of $MnCO_3$ typical for long-range translational motion of the crystal network. The 318.48 cm^{-1} band would correspond to bending modes (δ) Mn-O lattice vibration, thereby defining the carbonate unit integrity and metal–oxygen bonding environment. The 498.82 cm^{-1} peak could be attributed to in-plane bending vibrations of Mn–O stretching mode in $MnCO_3$. This vibrational mode indicates crystallization of $MnCO_3$ with minimal amorphous content. 644.46 and 898.31 cm^{-1} correspond to CO_3^{2-}, in-plane and out-of-plane bending (v_3, v_4) characteristic carbonate mode. 630.76 cm^{-1} peak is diagnostic of Mn-O stretching vibrations, reflecting partial surface oxidation which can contribute valuable pseudocapacitive characteristics. The prominent peak at 1078 cm^{-1} is associated with the symmetric stretching vibration (v_1) of the carbonate ion (CO_3^{2-}). This confirms that the incorporation of carbonate groups into the crystal lattice is successful. The 1551.9 cm^{-1} peak is indicative of the interaction of residual sugar on the surface due to Oswald's effect and the nano size of $MnCO_3$ sphere [1-4]. It has been found to increase electronic conductivity and electrochemical performance. Cumulatively, the Raman spectral features determine the phase-pure nature of $MnCO_3$ with minimal amounts of contribution of Mn-O bonding environments and carbonaceous morphology. These findings substantiate the suitability of the material for use as a supercapacitor where both electric double-layer capacitance (EDLC) and pseudocapacitive contribution are both essential.

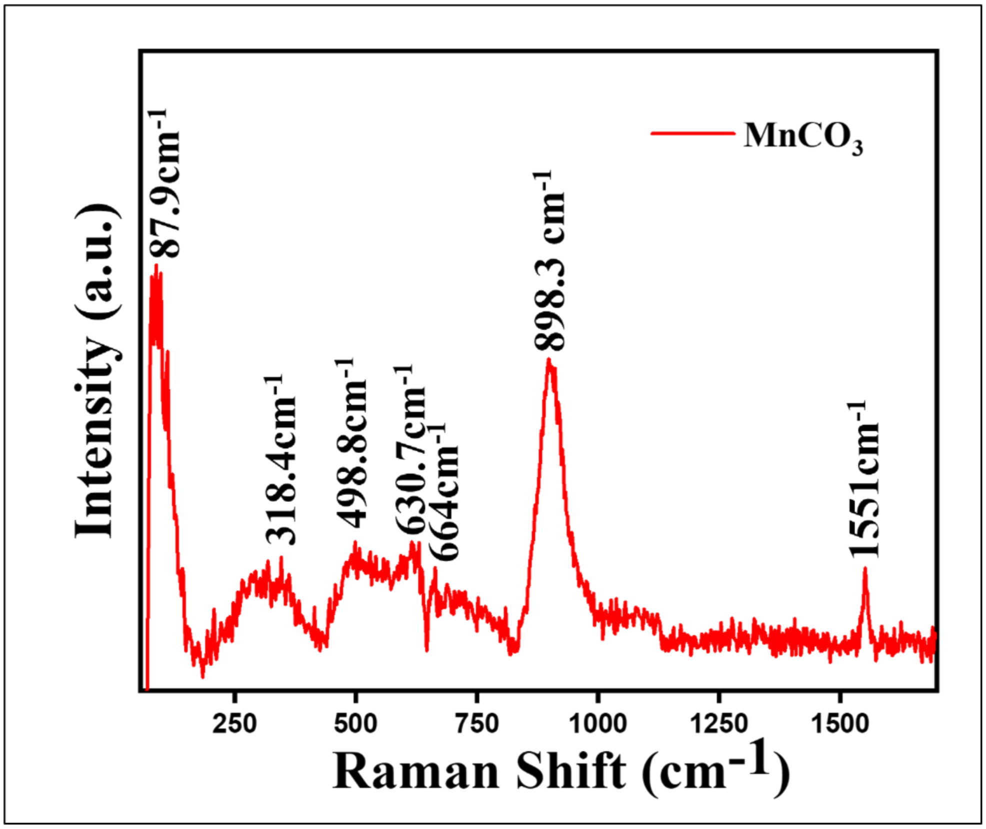

Figure S-2: Raman Spectra of $MnCO_3$

FTIR Analysis

Fourier Transform Infrared (FTIR) spectroscopy was employed to investigate the chemical structure and functional groups present in the synthesized $MnCO_3$ microspheres. The spectrum was recorded in the wavenumber range of 4000-500 cm^{-1}, as shown in Figure S-3. The FTIR profile reveals characteristic absorption bands that confirm the successful formation of manganese carbonate and provide insights into the molecular interactions within the material. Distinct vibrational bands corresponding to the carbonate (CO_3^{2-}) group were observed at 1366, 864, and 729 cm^{-1}. The strong band at 1366 cm^{-1} is attributed to the asymmetric stretching vibration of the CO_3^{2-} ions, while the peaks at 864 and 729 cm^{-1} correspond to out-of-plane and in-plane bending

modes, respectively. These features are consistent with the presence of the carbonate phase in the MnCO3 structure and are indicative of the successful synthesis of manganese carbonate.

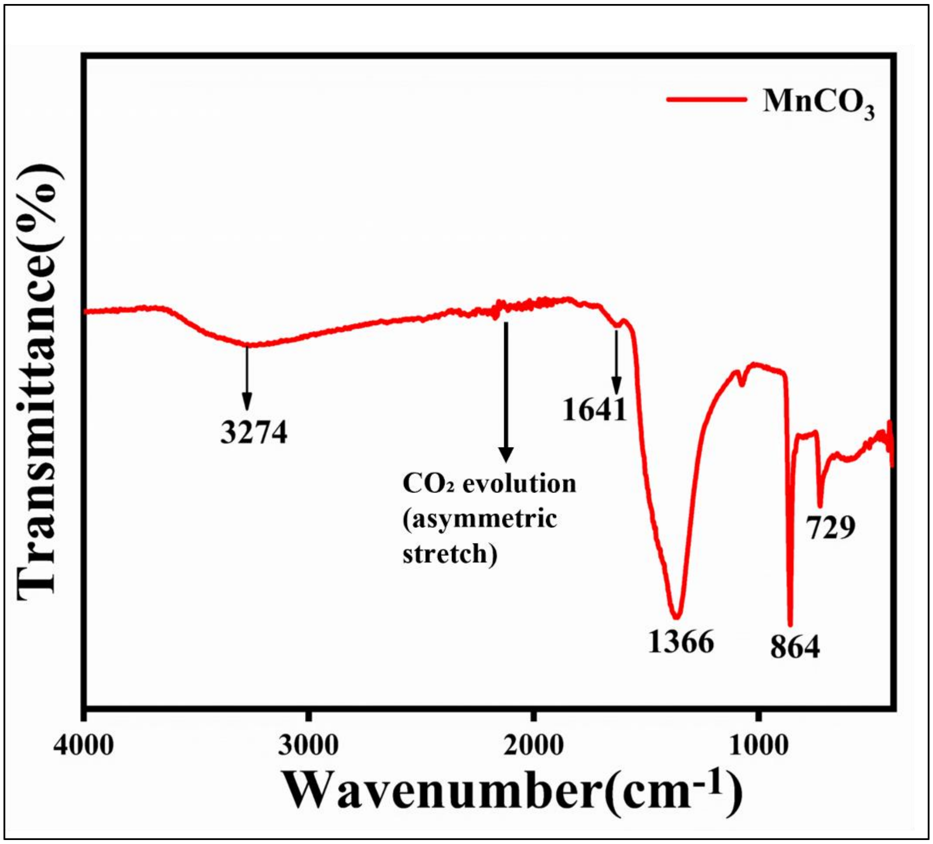

Figure S-3: FTIR Spectra of MnCO3 microsphere.

Additionally, a band at 1641 cm^{-1} is assigned to an overtone or combination band associated with CO_3^{2-} species interacting with Mn^{2+} cations. This interaction supports the incorporation of manganese into the carbonate matrix. A broad absorption band centered at approximately 3274 cm^{-1} is attributed to the O-H stretching vibration of physically adsorbed water molecules on the surface of the $MnCO_3$ microspheres. The presence of this band suggests some degree of surface hydration, which is common in carbonate materials exposed to ambient conditions. Overall, these FTIR features collectively validate the formation of $MnCO_3$ and indirectly support the proposed formation mechanism via CO_2 release and carbonate bonding. While we acknowledge the absence

of real-time pH or gas evolution monitoring in the current study, the spectroscopic evidence substantiates the chemical pathway proposed influencing the electrochemical behavior of the material, particularly in terms of ion transport and pseudo capacitance, making $MnCO_3$ a promising candidate for high-performance supercapacitor electrodes. Supercapacitor study of bare graphite sheet in three electrode system

As seen in Fig. S-4, the $MnCO_3$ electrode's electrochemical performance was evaluated in 2M KOH. The voltage range used to monitor the cyclic voltammetry (CV) was -1.0 to 0.7V. The cyclic voltammetry curves of bare and $MnCO_3$-loaded graphite sheet are displayed in Fig S-4a. The superior form of the cyclic voltammetry (CV) curves suggests a pseudocapacitive response for the $MnCO_3$ electrode material. This indicates that adding active material to the graphite sheet has enlarged its polygon area, which is an essential step in determining the capacitance using a CV plot. It should be mentioned that the coated graphite surface has a shifted potential window. A galvanostatic charge discharge test was conducted in order to verify the respective contributions of the loaded and naked graphite sheets. On the other hand, $MnCO_3$ had a 769 seconds discharge time at 0.7 A/g current density, but the discharge time for bare electrode was as low as 8.4 sec, as shown in Figure S-4b. This enormous variance indicates why adding $MnCO_3$ to graphite sheets is important and the changes it brought. A higher working potential was able to be attained demonstrating to us the graphite sheet's conductive properties. Figure S-4c sought to bring before us the influence of varying current density of bare graphite sheet, consequently having insignificant specific capacitance. These tests have shown us the significance of graphite sheets, which are flexible, chemically stable, and highly conductive.

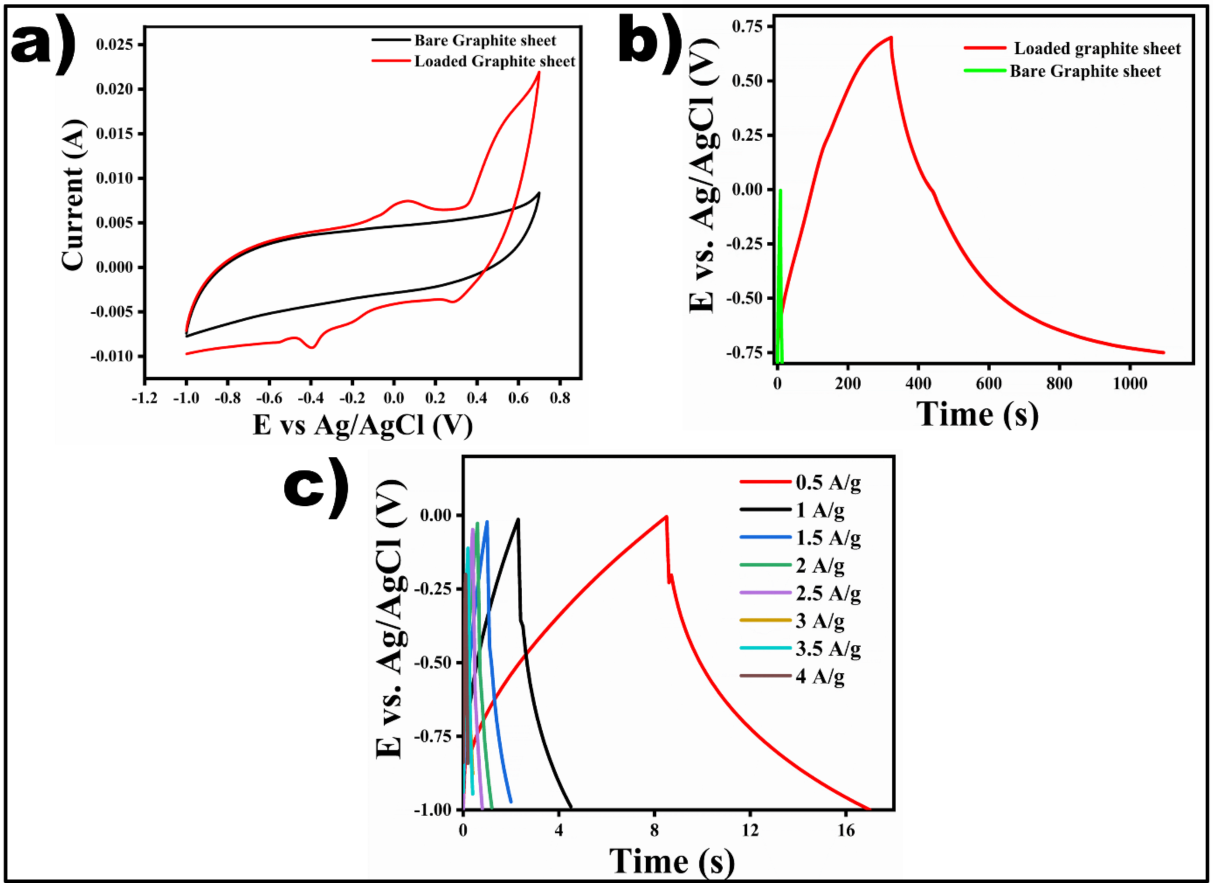

Figure S-4: Comparison study of Bare and $MnCO_3$ loaded graphite sheet in 2M KOH solution: a) Cyclic Voltammetry result at 50 mV/s. b) GCD plot for bare graphite sheet and $MnCO_3$ loaded sheet at 0.7 A/g c) GCD curves for bare graphite sheet at different current densities from 0.5 A/g to 4 A/g.

EIS Studies

To learn more about the ion transport and resistance of the $MnCO_3$ electrode in the 2M KOH electrolyte, electrochemical impedance spectroscopy was used. Figure S-5 displays the equivalent Nyquist plot, which was recorded at 5 mV amplitude and in the frequency range of 0.01 Hz to 100KHz for the before and after cycling data.

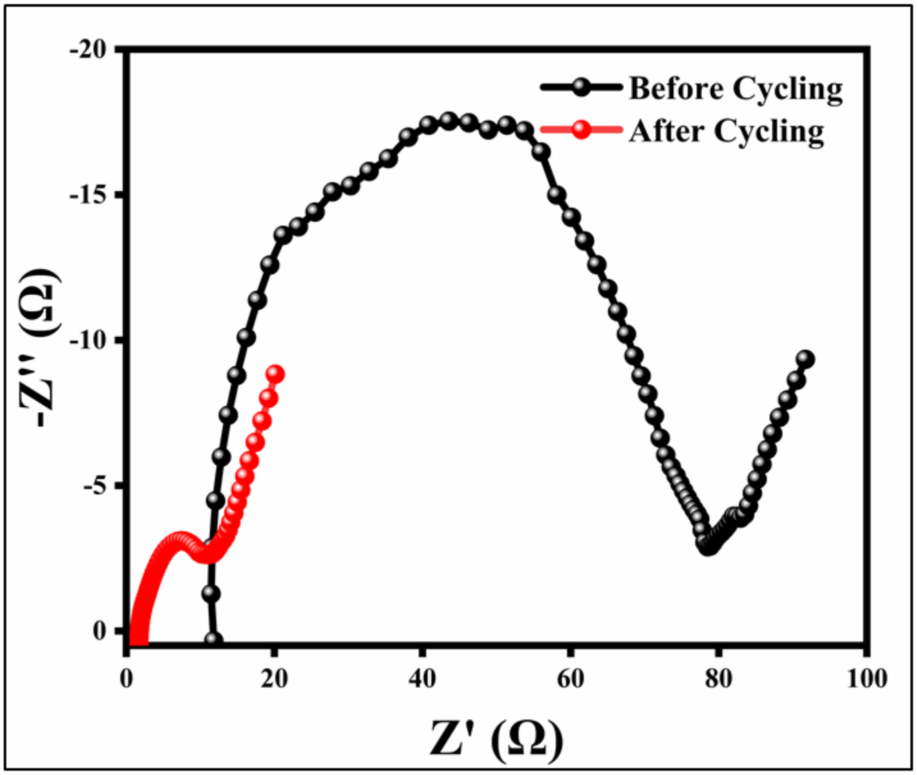

Figure S-5: EIS spectra for $MnCO_3$ electrode material before and after cycling

For investigations conducted after cycling as well as for pristine samples, the electrode's strong electrical conductivity and good reaction kinetics are confirmed by the EIS spectra, which showed a much lower R_{ct} value after cycling. It is made up of three distinct resistances: the electrolyte's resistance, the electroactive material's resistance, and the contact resistance between the electrode material and current collector of the semicircle in the second area (medium frequency). The third zone, known as the Warburg region, reflects the slope of the curve in the low-frequency region and is associated with the diffusion of redox species in the sample [5]. The electric double-layer development at the electrode–electrolyte interface is indicated by the vertical line in the low frequency region of each sample. Good contact between the current collector and

the electrode material is shown by the lack of the semi-circle in the medium frequency range. The corresponding R_{ct} value for before and after cycle is 67.39Ω and 9.44Ω respectively.

Previous publications state that due of the low ohmic drop and increased diffusion currents that provide complete access to the electrode's surface pores, high specific capacitance may be achieved at low current densities. In industrial applications, however, high current densities are important. The $MnCO_3$ sphere measuring conditions were carried out using higher current densities. The findings show that the measured specific capacitance values of 366 F/g at 0.7A/g for the $MnCO_3$ working electrode are greater than those previously reported for 3 electrode system.

Electrochemical charge storage mechanism

A significant advancement in understanding the charge storage kinetics in the $MnCO_3$ electrode is the detailed evaluation of their contributions. The intricate mechanism outlines the charge retention characteristics of $MnCO_3$ electrode and the methods employed for its analysis through cyclic voltammetry (CV) information at different sweep rates. The method of charge storage is defined by dividing it into three sections; [6] (i) Rapid electrochemical procedure: This includes the adsorption and desorption of charges. (ii) Faradaic component: This arises from charge-exchange activities involving surface atoms, referred to as pseudo capacitance, and (iii) non-faradaic component: This relates to the dual layer effect and the dispersive behavior of ions within the electrode substances and electrolytic solutions. Nevertheless, it is crucial to determine which mechanism primarily occurs in electrochemical reactions, therefore that we can readily grasp the charge storage process of the material for electrodes. We explored the diffusive and capacitive contributions from the CV data at different scan speeds, ranging from 5 mV/s to 100 mV/s. The connection between the peak current vs. various scan speeds has been employed for researching

the kinetic attributes of the electrode material [7,8]. At different scan rates, the power-law equation is applied as follows

$$i = a\ v^{b} \qquad (4)$$

by applying log on both sides, we can rewrite the above equation as

$$\log(i) = \log(a) + b \log(v) \qquad (5)$$

Here, v is the sweep rate, I is the anodic/cathodic peak current, and a and b are configurable parameters (b is constant). The constant parameter "b" is associated with the charge storage process. We calculate b value from the slope of the plot of log v vs log i. The reason for calculating b value is to determine the contribution of capacitive and diffusion supervised processes. There are 2 conditions significantly considered i.e, when b = 0.5 & b = 1.0. whilst b=1.0, the current response is taken proportional to scan rate whereas for b = 0.5 the current output is proportional to the square root of sweep rate being referred as diffusion-controlled process as shown in equation (6). Similarly, b value of 1 means capacitive behavior is dominant [9,10].

The b-values for the $MnCO_3$ were obtained by calculating the slope of the fitted curves between log(i) vs. log(v) in the surface-controlled capacitive and diffusion-controlled Faradaic processes, which are typically anodic and cathodic scan at different potentials. ent response presented a surface-controlled capacitive process dominance. It is observed that b values are not always in the vicinity of 1, even though every graph profile had b values ranging from 0.5 to 1, indicating both supercapacitor and battery features. The surface-controlled capacitive process, including the pseudocapacitive process, is responsible for contributions to the overall charge storage mechanism, as indicated by the b-values [11].

The capacitive and diffusion-controlled contributions to total capacity at a given potential at each scan rate were computed using the Dunn and his mates' approach.

$$i\,(\mathrm{V}) = \mathrm{k}_1\, \nu + \mathrm{k}_2 \sqrt{\nu} \qquad (6)$$

In this case, $\mathrm{k}_1\, \nu$ and $\mathrm{k}_2 \sqrt{\nu}$ stand for the currents from surface-controlled and diffusion-controlled processes, respectively, while i (V) indicates the current at a specific potential. An alternative form of the aforementioned equation is equation 7.

$$\frac{\mathrm{i\ (V)}}{\sqrt{\nu 1}} = \mathrm{k}_1 \sqrt{\nu 1} + \mathrm{k}_2 \qquad (7)$$

A linear representation of $i(\mathrm{V})/\sqrt{\nu}$ vs. $\sqrt{\nu}$, served to calculate the k_1 and k_2 values at different scan rates [6,7].

Table S-1: **Electrochemical studies comparison of $MnCO_3$ and $MnCO_3$ based composites for supercapacitor application**

Materials	Electrolyte & Molarity	Potential Window (V)	Scan Rate	Specific Capacitance	Cycle No and Retention	Reference
$MnCO_3$	0.1M NA_2SO_4	0-1V	0.5 A/g	194F/g	10,000 & 92%	5
$MnCO_3$	0.1M $Mg(ClO_4)_2$	0-1V	0.34 A/g	144F/g	1000 &	14
$MnCO_3$	0.1M $Mg(ClO_4)_2$	0-1V	1 mA	216F/g	500 & 90.3%	15

$MnCO_3$	0.1M $Mg(ClO_4)_2$	0-1V	0.5mA	296F/g	500& 81.1%	16
$MnCO_3$@ MnO_2	1M Na_2SO_4	-0.2-0.8V	1A/g	363 F/g	2000& 84.2%	17
$MnCO_3$	0.1M $Mg(ClO_4)_2$	0-1V	0.33A/g	188F/g	10,000&92%	18
$MnCO_3$	6M KOH	0-0.4V	0.5A/g	163.7F/g	1000 & 91%	19
$MnCO_3$	0.1M $NaClO_4$	0-0.9V	0.15A/g	129F/g	1000& 87%	20
**$MnCO_3$**	**2M KOH**	**-1 -0.7V**	**0.7A/g**	**366 F/g**	**3000 & 98.3% @4.2 A/g**	**This work**
**$MnCO_3$**	**2M KOH**	**0- 1.2V**	**0.5 mA**	**179.8 F/g**	**10000 & 99.6%**	**(SSC device) This work**

Post Cycling Performance for $MnCO_3$ after 3000 Cycles in a three-electrode system

To scrutinize the structural integration of $MnCO_3$ after long electrochemical cycling, ex-situ X-ray diffraction (XRD) was performed after charge-discharge cycles exhibiting prominent peaks at 2Θ = 26.4° and 54.7° corresponding to (002) and (004) planes of graphite (JCPDS 00-008-0415) due to graphite sheet being used as current collector in the three-electrode system. Because the intensity of these graphite sheets is high, the characteristic $MnCO_3$ peaks are supressed. The enlarged XRD plots are added in the inset of the main XRD pattern. The reflections at at 2θ values of 23.9°, 31.8°, 34.2°, 36.5, 49.2°, 50.9°, 52.6° corresponds to (012), (104), (006),

(110), (024), (018), and (116) planes respectively. Even beyond 3000 cycles, the $MnCO_3$ electrode retains its crystallographic integrity and phase purity, indicating its exceptional structural durability under demanding electrochemical circumstances, as evidenced by the lack of any secondary or impurity phases in the XRD pattern. (Figure S-6)

We also conducted post-cycling FESEM analysis of the $MnCO_3$ electrode after long-term cycling. As shown in the Fig. S-7, the post-cycling electrode is, a petal like morphology in contrast to the spherical morphology observed before cycling. This implies that a structural reconstruction occurs during the charge -discharge process, leading to the formation of nanosheets that provide a larger surface area and more active sites, facilitating enhanced ion transport and redox activity. This confirms the excellent mechanical integrity and surface stability even after prolonged cycling. Elemental mapping delivers a uniform distribution of all the constituent elements. The subsequent EDAX further reveals the dominance of Mn, C and O with no signs of decomposition or impurity, reasserting long term chemical stability.

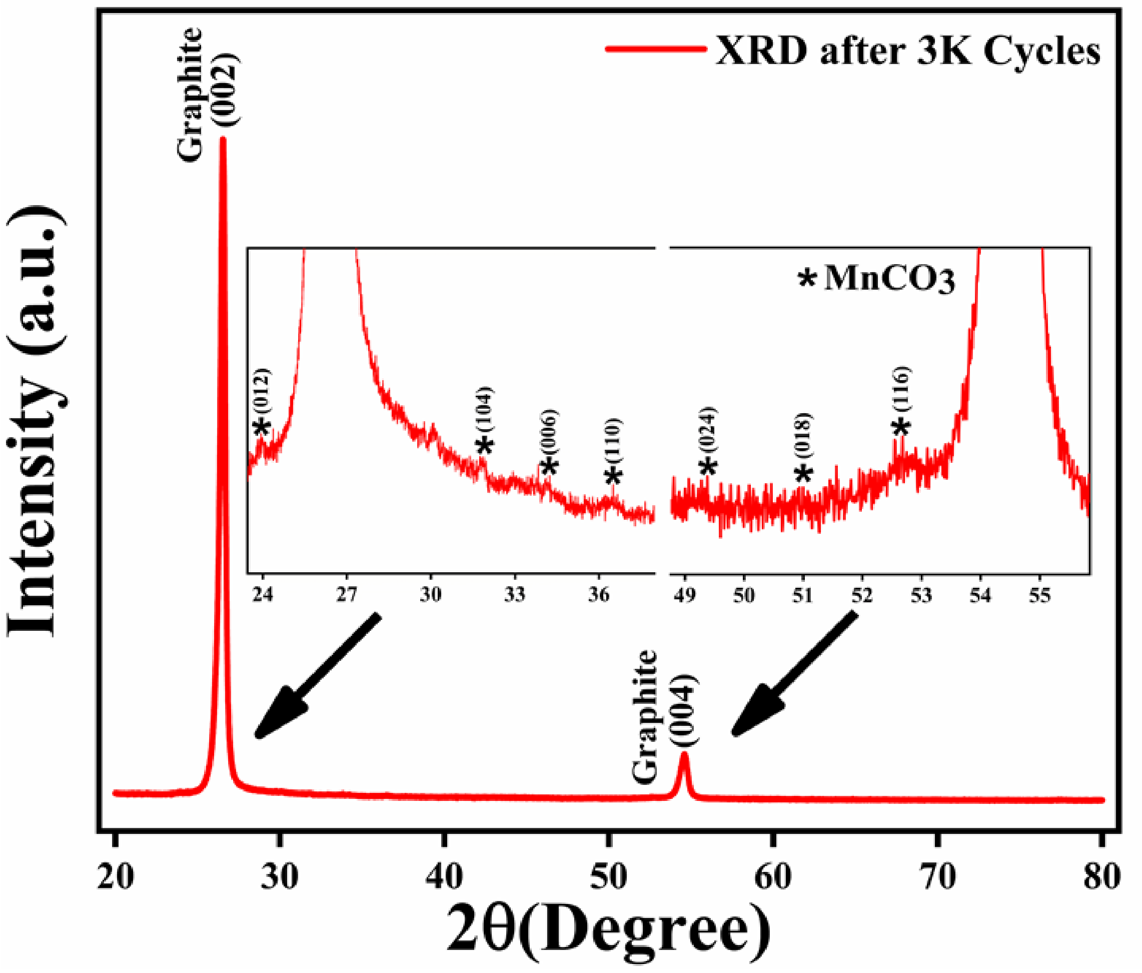

Figure S-6: Post cycling ex-situ-XRD analysis of $MnCO_3$ after 3000 cycles for three electrode system.

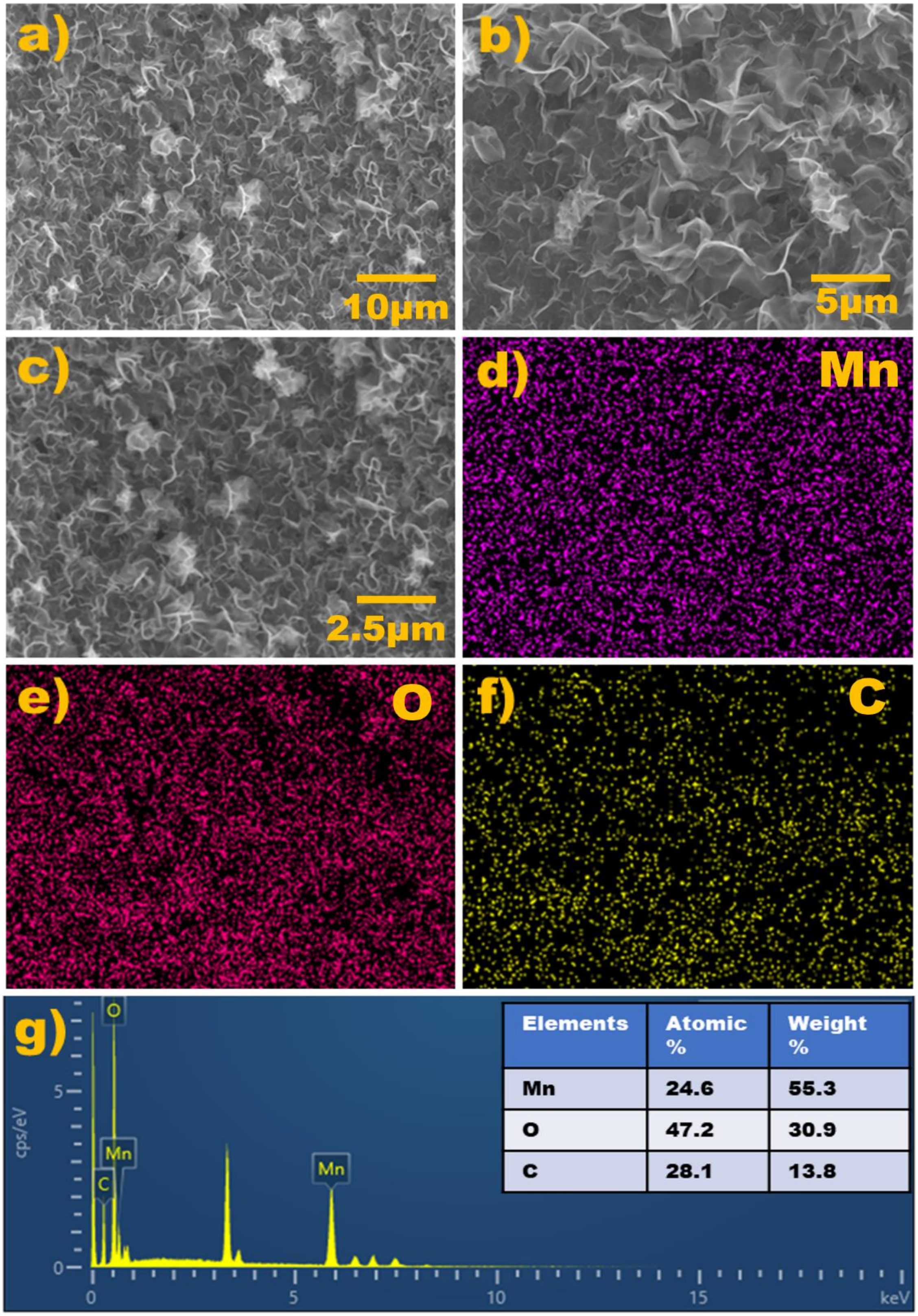
a)
10μm
b)
5μm
c)
2.5μm
d)
Mn
e)
O
f)
C
g)
cps/eV
keV
Elements
Atomic %
Weight %
Mn 24.6 55.3
O 47.2 30.9
C 28.1 13.8

Figure S-7: Post-cycling FESEM Images $MnCO_3$. a) 10µm, b) 5 µm, c) 2.5µm, Elemental mapping analysis (c), d) Mn, e) O, f) C, EDAX spectrum (inset: Elemental weight and atomic percentage of all elements).

Reference

[1] Komura, H., Akiyama, H., Fukaya, M., & Ishikawa, K. Raman scattering from phonons in $MnCO_3$. Journal of Raman Spectroscopy, 1983,14 (5), 330–332.

[2] Wang, C.; Ren, L.; Walters, J. B.; Zhang, L.; Tao, R. In situ Raman vibrational spectra of siderite ($FeCO_3$) and rhodochrosite ($MnCO_3$) up to 47 GPa and 1100 K. American Mineralogist, 2023, 108(1), 1–9.

[3] Devaraj, S.; Liu, H. Y.; Balaya, P. $MnCO_3$: A novel electrode material for supercapacitors. Journal of Materials Chemistry A, 2014, 2(12), 4276–4281.

[4] Wang, C.; Ren, L., Walters, J. B., Zhang, L., & Tao, R. In situ Raman vibrational spectra of siderite ($FeCO_3$) and rhodochrosite ($MnCO_3$) up to 47 GPa and 1100 K. American Mineralogist, 2023, 108(1), 1–9.

[5] Vardhan, P.V.; Idris, M.B.; Ramanathan, V.; Devaraj, S. Electrodeposited $MnCO_3$ as a High-Performance Electrode Material for Supercapacitor. Wiley, 2018, 3, 6775-6778.

[6] Cai, Z.; Zhang, F.; We, D.; Zhai, B.; Wang, X.; Song, Y. $Ni_xCo_{1-x}S_2$@N-doped carbon composites for supercapacitor electrodes. Journal of Energy Storage. 2023, 72, 108231.

[7] Michael, A.; Woung, J.; Sarah, H.; Héctor, D.; High, B.; Augustyn, V.; Come, J.;. Lowe, M. A.; Kim, J.W.; Taberna, P.; Tolbert, S.H.; Abruña, H.D.; Simon, P.; Dunn B.

High-rate electrochemical energy storage through Li^+ intercalation pseudo capacitance. Nature Materials, 2013, 12, 518 522.

[8] Gandla, D.; Zhang, F.; Tan, D.Q. Advantage of Larger Interlayer Spacing of a $Mo_2Ti_2C_3$ MXene Free-Standing Film Electrode toward an Excellent Performance Supercapacitor in a Binary Liquid – Organic Electrolyte. ACS Omega, 2022, 7, 7190−7198.

[9] Kumar, R.; Bhuvana, T. A. Sharma; Ammonolysis Synthesis of Nickel Molybdenum Nitride Nanostructure for High-performance Asymmetric Supercapacitor. New J. Chem., 2020, 44, 14067-14074

[10] Tan, Y.; Meng, L.; Wang, Y.; Dong, W.; Kong, L. Negative electrode materials of molybdenum nitride / N-doped carbon nano- fiber via electrospinning method for high-performance supercapacitors. Electrochimica Acta, 2018, 277, 41–49.

[11] Wang, L.; Zhao, T.; Chen, R.; Fang, H.; Yang, Y.; Cao, Y.; Zhang, L. Molybdenum nitride and oxide quantum dot@nitrogen-doped graphene nanocomposite material for rechargeable lithium-ion batteries. Batteries, 2023, 9, 32.

[12] Li, W.; Fukunishi, M.; Morgan, B.J.; Borkiewicz, O.J.; Chapman, K.W.; Pralong V.; Maignan, A.; Lebedev, O.I.; Ma, J.; Groult, HKomaba, .; S.; Dambournet, D. A reversible phase transition for sodium insertion in anatase TiO_2. Chem. Mater. 2017, 29, 1836–1844.

[13] Le, Z.; Liu, F.; Nie, P., Li, X.; Liu, X.; Bian, Z.; Chen, Wu, H. B.; Lu, Y. Pseudocapacitive sodium storage in mesoporous single-crystal-like TiO_2-graphene nanocomposite enables high-performance sodium-ion capacitors. ACS Nano, 2017, 11, 2952–2960.

[14] Vardhan, P. V.; Sridhar, S.; Sivakkumar, S.; Mudali, R.; Kamachi, U.; Devaraj, S. Facile Synthesis of Mesoporous $MnCO_3$ for Supercapacitor Applications. Journal of Nanoscience and Nanotechnology, 2018, 18, 2775-2780

[15] Devaraj, S.; Liu, H.Y.; Balaya, P.; $MnCO_3$: a novel electrode material for supercapacitors. Journal of Materials Chemistry A, 2014, 2 4276-4281

[16] Devaraj, S.; Vardhan, P.V.; Liu, H.Y.; Balaya, P. Metal carbonates: alternative to metal oxides for supercapacitor applications? A case study of $MnCO_3$ vs MnO_2. Journal of Solid-State Chemistry, 2016, 20, 1877-1883.

[17] Chen, H.; Yan, Z.; Liu, X. Y.; Guo, X.L.; Zhang, Y. X.; Liu, Z.H. Rational Design of Microsphere and Microcube $MnCO_3$@MnO_2 Heterostructures for Supercapacitor Electrodes. J. Power Sources, 2017. 353,202−209.

[18] Palem, V.V. Ultrasonic-assisted, rapid preparation of mesoporous $MnCO_3$ for electrochemical supercapacitor applications: A novel approach. Journal of Electroanalytical Chemistry, 2023,942, 117571.

[19] Peng, H.H.; Zhang, L.; Chen, J. Facile synthesis of MnCO3 nanoparticles on Ni foam for binder-free supercapacitor electrodes. International Journal of Electrochemical Science, 2017, 12, 5898-5909.

[20] Zhang, N.; Ma, J.; Li, Q; Li, J.; DHL Ng; Shape-controlled synthesis of $MnCO_3$ nanostructures and their applications in supercapacitors. RSC Advances, 2015, 5, 81981-81985.

[21] Hu H, Xu JY, Yang H, Liang J, Yang S, Wu H. Morphology-controlled hydrothermal synthesis of MnCO3 hierarchical superstructures with Schiff base as stabilizer. Mater Res Bull 2011;46(11):1908–15.

[22] Udayabhanu U, Muralikrishna S, Kishore B, Nagabhushana H, Suresh D, Sharma SC, et al. One pot green synthesis of $MnCO_3$-rGO composite hybrid superstructure: application to lithium-ion battery and biosensor. New J Chem 2017;41(21):12854–65.
